# Improved Removal Capacity and Equilibrium Time of Maghemite Nanoparticles Growth in Zeolite Type 5A for Pb(II) Adsorption

**DOI:** 10.3390/nano10091668

**Published:** 2020-08-26

**Authors:** Juan A. Ramos-Guivar, Katterine Taipe, Miguel Angelo Schettino, Eloi Silva, Marco Antonio Morales Torres, Edson Caetano Passamani, Fred Jochen Litterst

**Affiliations:** 1 Departamento de Física del Estado Sólido (DAFES), Facultad de Ciencias Físicas, Universidad Nacional Mayor de San Marcos, P.O. Box 14-0149, Lima 14, Peru; katterine.taipe@unmsm.edu.pe; 2 Department of Physics, Federal University of Espírito Santo-UFES, Vitória, ES 29075-910, Brazil; miguel.ufes@gmail.com (M.A.S.J.); passamaniec@yahoo.com.br (E.C.P.); 3 Department of Chemistry, Federal University of Espírito Santo-UFES, Vitória, ES 29075-910, Brazil; eloi.silva@ufes.br; 4 Department of Theoretical and Experimental Physics, Federal University of Rio Grande do Norte-UFRN, Natal, RN 59078-970, Brazil; marco.moralestorres@gmail.com; 5 Institut für Physik der Kondensierten Materie, Technische Universität Braunschweig, 38110 Braunschweig, Germany; j.litterst@tu-bs.de; 6 Centro Brasileiro de Pesquisas Físicas, Rio de Janeiro, RJ 22290-180, Brazil

**Keywords:** maghemite, zeolite type 5A, lead removal, water treatment

## Abstract

Novel magnetic zeolite type 5A nanocomposites were synthesized by the co-precipitation method and applied to lead removal from aqueous ambient. Maghemite nanoparticles were mixed with zeolite and, by controlling its content, transmission electron microscopy results gave sizes of 5 to 15 nm and selected area electron diffraction patterns confirmed the presence of zeolite. The nanocomposites have high specific surface area with values up to 194 m^2^/g. Magnetization measurements proved superparamagnetic behavior with saturation values of ~35 emu/gFe. Kinetic adsorption experiments showed removal efficiencies of 99.9% and an enhanced equilibrium time of 5 min. The lead concentrations after adsorption experiments lay under the permissible levels of 10 μg L^−1^, according to the World Health Organization. The maximum adsorption capacity, estimated by Sips model, was 265 mg L^−1^ at 300 K. The removal efficiency was significantly improved in the range of pH > 6, as well as in the presence of cation interferents such as Ca(II), Cu(II), and Cd(II). The adsorption mechanism was explained with cation exchange between Pb(II), the zeolite framework, and the protonated maghemite surface. Besides, our system revealed recyclability even after seven regeneration cycles. Thus, our synthesized materials have remarkable adsorption properties for lead water remediation processes.

## 1. Introduction

Polluted water often presents non-biodegradable effluents like heavy metals and organic materials, which are toxic and can cause several illnesses [1,2]. Nowadays, drinking water is systematically contaminated, for example, by several heavy metals from different sources. Contamination of water with lead (Pb(II)) may be the result of the water contact with household plumbing systems that contain lead devices; bad manipulation of lead-acid batteries; disasters in mining companies; and, of course, illegal mining [3]. The permissible concentration level of lead in water is 10 μg L^−1^ according to World Health Organization (WHO) guidelines [3]. Excess ingestion can provably cause health problems like mental retardation as well as kidney and brain diseases [3,4]. Therefore, in this current context, several lead separation methods (e.g., electrochemical treatments, membrane filtration, and chemical precipitation) have been developed and applied to improve the water quality from different sources [5]. Among these methods, the adsorption process is highly advantageous owing to its reproducibility, cost-effective implementation, and relative simplicity in possible commercial applications [6].

One of the most suitable materials for application in the adsorption process is zeolites, having interesting catalytic and adsorbent properties [7,8]. This class of materials is composed of crystalline aluminum-silicate and alkali earth elements like sodium and calcium [7], keeping the stoichiometric formula $M_{\frac{x}{n}}\left[{\left({\mathrm{AlO}}_{2}\right)}_{x}{\left({\mathrm{SiO}}_{2}\right)}_{y}\right]zH_{2}O$, where *x* and *y* are integer numbers, *n* represents the valence of cation M, and *z* is related to the number of water molecules in the unit cell. They can be divided into natural and synthetic zeolites. Among all-natural families of zeolites, 40 species are found [8]. In addition, there exist about 150 types of synthesized zeolites, which are designated with a specific letter: Type A, Type X, Type Y, Type ZSM, and so on [8]. They differ in the pore size, specific surface area, and positive cations that balance the molecules and influence the pore diameters and adsorptive properties [8]. Moreover, it should be said that zeolites have been used as adsorbents for organic and metal pollutants [7,9]. In general, they have demonstrated excellent cation-exchange selectivity for heavy metals owing to their featured porous properties and chemical composition [7].

On the other hand, Fe-oxide based nanoadsorbents present mesoporous and chemical surface configurations that can also favor the adsorption of metal cations [10]. Additionally, iron oxide nanoparticles (NPs) are low-cost and environmentally friendly materials. Thus, these Fe-oxide NPs can be combined with other adsorbents to improve the adsorption and magnetic separation processes [11]. For instance, systems based on magnetic nanoparticles coated montmorillonite [12], core-shell gold coated magnetic nanoparticles [13], iron-based nanoparticles–zeolite composites [14], zero valent iron [15], Co-doped Fe_2_O_3_ [16], MNPs@carboxymethylated biopolymers [17], and the composite of solid matrices with iron oxides have been proposed as good candidates for bioremediation processes [18]. To our knowledge, however, a magnetic nanocomposite made of maghemite and zeolite type 5A has not been studied and tested for lead removal. So, in this work, we have prepared ferromagnetic zeolite composites using maghemite NPs as precursor that were mixed with mesoporous zeolite type 5A, controlling their contents. Synthesis experiments were performed to obtain the optimum material for Pb(II) adsorption and to be magnetically removed with an applied field. The crystal structure, sample surface, and their magnetic properties were systematically studied by X-ray diffraction, N_2_ adsorption–desorption, and vibrating sample magnetometry (VSM) measurements. Their application as magnetic adsorbents was tested for Pb(II) removal from synthetic aqueous solution up to lead concentrations reaching value below the quantification limits of inductively coupled plasma atomic emission spectroscopy (ICP-OES). We conclude that combining zeolite adsorption and magnetic separation properties facilitates the removal of lead from contaminated water easily, making our nanocomposite a promising material that is suitable for water remediation in effluents with high lead contents.

## 2. Materials and Methods

### 2.1. Materials and Chemicals

The following chemical compounds of analytical grade, obtained from Sigma Aldrich (São Paulo, Brazil), were used without further purification: iron sulfate hepta hydrated (FeSO_4_·7H_2_O), iron(III) chloride anhydrous (FeCl_3_), ammonia hydroxide (NH_4_OH, 28–30%), and zeolite type 5A. Lead nitrate, Pb(NO_3_)_2_, from Dinâmica (São Paulo, Brazil), was used.

### 2.2. Adsorbent Synthesis

The co-precipitation method is suitable to obtain high quantities in term of mass for desired industrial scalability. It consists in mixing iron precursors (Fe(II)/Fe(III) = 0.5 ratio, an optimum ratio that favors spherical NPs in high alkaline medium (pH = 10–12). It should be also mentioned that (i) solid separation and washing procedures are easy to handle using magnetic separation protocol and (ii) the zeolite content was modified in the synthesis to obtain the optimum nanoadsorbent for Pb(II) adsorption. Specifically, we prepared zeolite type 5A composites using three different synthesis processes, which are individually described below.

#### 2.2.1. Synthesis 1

An amount of 0.5 g of the zeolite type 5A was dispersed with distilled water (50 mL) in a glass cylinder container (beaker) and left to stir for 30 min. Then, 6 g of FeCl_3_ (37 mmol) and 5.1 g of FeSO_4_·7H_2_O (18.5 mmol) were added to this dispersion. Immediately after the first step, a solution of NaOH (1.5 M) solution was dropped to the mixture till a black color covered the total solution, as a result of NPs formation. The pH of the solution was set to 10. The chemical reaction was left at 80 ± 2 °C, for 6 h. After that, we cooled the reaction and the material was washed several times and separated with the help of magnetic decantation. This sample was labeled as NPZEO1.

#### 2.2.2. Synthesis 2

A weighted amount of 0.5 g of the zeolite type 5A was dispersed in distilled water (50 mL) for 30 min under vigorous stirring. In sequence, 6 g of FeCl_3_ (37 mmol) and 5.1 g of FeSO_4_·7H_2_O (18.5 mmol) were added to the previous dispersion. A Fe(II)/Fe(III) molar ratio equal to 0.5 was used (spherical particles were expected under this condition). Subsequently, NH_4_OH (30%) was added dropwise to the dispersion until the value of pH has reached 10. A black precipitate formed as a characteristic of iron oxide NPs formation in alkaline medium. The chemical reaction was left at 80 ± 2 °C, for 6 h. After cooling the reaction, the material was washed several times and, using magnetic decantation, it was separated. This sample was labeled as NPZEO2.

#### 2.2.3. Synthesis 3

An amount of 2.5 g of the zeolite type 5A was dispersed in distilled water (300 mL) in a beaker for 30 min under vigorous stirring. After that, 1.6 g of γ-Fe_2_O_3_ NPs prepared by co-precipitation (same conditions as synthesis 1) was added in the previous dispersion at room temperature (RT), for 24 h. The pH of the dispersion was neutral. Thereafter, the sample was filtered with a 2 μm membrane filter to remove the excess of NPs that had not precipitated or/and interacted with the zeolite matrix. Finally, the sample was dried in an oven for 36 h. We refer to this sample as NPZEO3.

### 2.3. Characterization of Structural, Surface Area, and Magnetic Properties

The X-ray diffraction (XRD) data were collected using a Bruker D8 diffractometer, operating with Cu Kα radiation (1.5406 Å), at 50 kV and 100 mA. Powder X-ray diffraction (XRD) patterns were obtained in step scanning mode, 2θ = 8–80° with a step of 0.01°. The crystallographic information file (CIF) given by the software Match V3 (version 3, Crystal Impact, Bonn, Germany) [19] was #9006316 for maghemite and #9002158 for goethite. The γ-Fe_2_O_3_ crystallographic parameters obtained by Pecharroman et al. [20] were used as initial refinement parameters (cubic, space group Fd$\bar{3}$m, a = 8.33 Å). For goethite, the crystallographic parameters obtained by Gualtieri were used (orthorhombic, space group Pnma, a = 9.91 Å, b = 3.01 Å, c = 4.58 Å) [21]. The CIF for the zeolite was #2102130, the space group was F$\bar{4}$3c, cubic, and a = 24.5 Å [22]. For the quantitative analysis in the composites, the scale factor was related to the weight fraction of each crystallographic phase, W_j_ [23]. The degree of crystallinity (DOC) was estimated from the XRD pattern according to the following [24]:

$$DOC\;\left(\%\right)=\frac{Area\;of\;crystalline\;peaks\;}{Area\;of\;\left(crystalline+amorphous\right)\;peaks\;}\times 100$$

Transmission electron microscopy (TEM) images and selected area electron diffraction (SAED) patterns were obtained using a JEOL-JEM2100 microscope (Tokyo, Japan). Nitrogen (N_2_) adsorption–desorption measurements isotherms at 77 K were measured with Autosorb-1 equipment. Before the recording of the data, the samples were degassed at 130 °C for 6 h. Then, the textural properties, including surface specific area (SSA) and pore size distribution (PSD), were determined by employing a multi-Brunauer–Emmett–Teller (BET) point plot and the non-local density functional theory (NLDFT) model [25]. This last method is suitable for the applicable pore diameter range of 0.35 to 35 nm. The carbon kernel file at 77 K, based on a slit-pore model and on a cylindrical pore model, was used to obtain the pore size distribution of the functionalized NPs. The physical properties measurement system (PPMS Dynacool, Quantum Design Latin America, San Diego, CA, USA) equipped with a vibrating sample magnetometer from quantum design was used to obtain magnetic hysteresis (M-H) loops at RT and 5 K. Zero field cooling (ZFC) and field cooling (FC) protocols were done to record magnetization data in the temperature range from 5 to 300 K under a probe field of 70 kOe.

### 2.4. Heavy Metal Adsorption Experiments

The synthetic aqueous solutions of lead were prepared from Pb(NO_3_)_2_. The adsorption kinetic experiments, influence of Pb concentration, and adsorbent mass were conducted at pH = 6. The batch kinetic experiments were carried out by stirring 25 mg of adsorbent with 50 mL of a 50 mg L^−1^ initial Pb(II) solution at 25 °C for intervals of 5 min to 4 h. After finding the equilibrium adsorption time, the adsorption isotherm was obtained from an initial Pb(II) solution of 130 mg L^−1^. Subsequent dilutions were done to obtain the total isotherm, keeping the same dosage of 0.5 g L^−1^. The adsorbent dose was tested by varying the adsorbent amount (5, 10, 15, 25, and 50 mg) in contact with 50 mL of Pb(II) solution. The batch adsorption experiments were carried out under continuous shaking at 150 rpm and at RT, for 1 h. For speed agitation experiments, speed ranges of 50 to 500 rpm were employed. An adsorbent mass of 25 mg and an initial volume of 50 mL were used at RT. For the determination of the pH dependence of the adsorbed amount, an initial concentration of 70 mg L^−1^ was used, and the pH was changed using HCl (0.01 M) and NaOH (0.1 M) solutions. The initial pH slightly differs from the equilibrium one (5.8–5.9), but, in first approximation, it can be assumed to be equal to 6 for the experiments. The pH equilibrium value was measured in all the experiments. The quantification of lead concentrations was done by ICP-OES (inductively coupled plasma atomic emission spectroscopy). A Perkin Elmer Optima 7000 DV spectrometer (Waltham, MA, USA) was used for measurements after calibration with stock solutions. The adsorbed lead amount was calculated by the difference between the initial ($C_{0}$) and final $\left(C_{t}\right)$ concentrations in solutions. The adsorbed amount is defined as follows:

(1) $$q_{t}=\frac{\left(C_{0}-C_{t}\right)V}{m}\;(\mathrm{mg}\;g^{-1})$$

where $q_{t}$ is the amount, in mg, of adsorbate adsorbed per gram of adsorbent for a certain time *t*; the parameter *m* is the adsorbent mass (mg); and *V* (mL) is the reservoir volume used for batch adsorption tests.

To compare the kinetic and isotherm adsorption models, we used the Akaike’s information criterion (AIC). The best fit to the experimental data has the lowest value of AIC [26].

(2) $$AIC=Nln\left(\frac{SSE}{N}\right)+2N_{p}+\frac{2N_{p}\left(N_{p}+1\right)}{N-N_{p}-1}$$

where *N* corresponds to the number of experimental data, *N_p_* is the number of parameters in the respective model, and SSE is the sum square error given by the following:

(3) $$SSE=\overset{t}{\underset{t=0}{\sum }}{\left(q_{t,exp}-q_{t,model}\right)}^{2}$$

where $q_{t,exp}$ and $q_{t,model}$ are the adsorption capacities, respectively, calculated from the experimental data at the equilibrium time and from the respective kinetic and isotherm models.

## 3. Results and Discussion

### 3.1. Structural and Morphological Properties

Several phenomenological models were systematically applied to study the microstructural parameters obtained from XRD patterns of the samples. In this sense, it is important to mention that the physical information of the XRD pattern of a particular sample must be firstly corrected using the instrumental broadening correction, according to Equation (4) below:

(4) $$\beta_{D}^{2}=\left[{\left(\beta^{2}\right)}_{\mathrm{measured}}-{\left(\beta^{2}\right)}_{\mathrm{inst}}\right]$$

where $\beta_{D}$ corresponds to instrument-corrected broadening of the XRD pattern.

Thus, the simplest and most common method to estimate the average crystalline grain sizes is the Scherrer’s formula, given by the following:

(5) $$D=\frac{k\lambda }{\beta_{D}\cos\theta }$$

where k = 0.94 nm^−1^ for spherical shape morphologies [27], λ corresponds to Cu K*α* radiation’s wavelength, $\beta_{D}$ is peak broadening (Equation (4)), and *θ* is the angular peak position. Then, a linear plot of cos*θ* versus 1/$\beta_{D}$ can be fitted and the slope of the curve is related to the average grain size, as shown by the results in Figure S1 (Supplementary Material). The calculated grain size values are reported in Table S1. The zeolite type 5 A has an average grain size of 66 nm, the maghemite in the NPZEO1 sample has a grain size of 11.2 nm, and the goethite an average size of 58.9 nm. The NPZEO2 sample presents only one crystalline phase of maghemite with an average size of 16.8 nm, while the NPZEO3 sample displays a presence of two phases: zeolite type 5A with grains of 112.3 nm and maghemite with average sizes of 7.8 nm.

On the other hand, the Scherrer’s estimation does not consider the internal strain in the crystalline grains provoked by crystal imperfections and local distortions of the lattice. However, the strain parameter is included in the Williamson–Hall’s (W-H) method [27]:

(6) $$\beta_{hkl}cos\theta =\left(\frac{k\lambda }{D}\right)+\left(4\varepsilon sin\theta \right)$$

where ε (%) is the induced strain of crystalline lattice. Equation (3) represents the uniform deformation model (UDM), which assumes the strain uniform in all crystallographic directions [27]. By contrary, the uniform strain deformation model (USDM) is a more generalized perspective, as it includes Hooke’s law and refers to strain in the W-H by introducing σ=Yε, where σ is the crystal’s stress and the quantity *Y* is the Young’s modulus. So, we can rewrite Equation (6) as follows:

(7) $$\beta_{hkl}cos\theta =\left(\frac{k\lambda }{D}\right)+\left(\frac{4\sigma sin\theta }{Y_{hkl}}\right)$$

For a cubic crystal, the Young’s modulus can be calculated using the following [28]:

(8) $$Y_{hkl}=\frac{9B_{0}G_{V}}{3B_{0}+G_{V}}$$

where

(9) $$G_{V}=\frac{1}{5}\left(3C_{44}+2C^{\prime }\right)$$

(10) $$C^{\prime }=\frac{1}{2}\left(C_{11}-C_{12}\right)$$

(11) $$C_{11}+2C_{12}=3B_{0}$$

are the elastic constants of cubic crystals and the constants $C_{44}=71.0$, $C_{11}=380.0$, and $C_{12}=257.0$ GPa were obtained from computational calculations [28]. The value obtained for $Y_{hkl}$, by substituting Equations (9)–(11) into Equation (8), is 187.5 GPa (this value will be substituted into Equation (7) or (13)). On the other hand, the uniform deformation energy density model (UDEDM) takes into account the following [27]:

(12) $$\beta_{hkl}cos\theta =\left(\frac{k\lambda }{D}\right)+\left(4sin\theta {\left(\frac{2U}{Y_{hkl}}\right)}^{1/2}\right)$$

where the energy density, U, is defined as $U=\frac{\left(\varepsilon^{2}Y_{hkl}\right)}{2}$, where σ=εY can be expressed by the following:

(13) $$U=\frac{\left(\varepsilon^{2}Y_{hkl}\right)}{2}=\left(\frac{\sigma^{2}}{2Y_{hkl}}\right)$$

**Figure 1:**
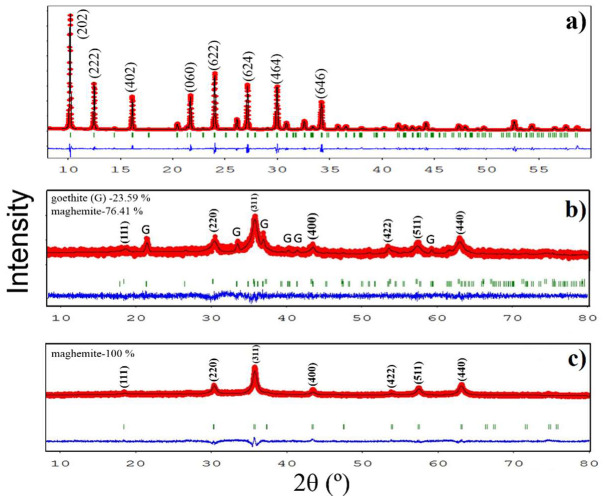
X-ray diffraction (XRD) patterns with their Rietveld refinements for zeolite type 5A (**a**), NPZEO1 (**b**), and NPZEO2 (**c**) samples. The observed experimental diffractograms are given by the red lines (I_obs_), the black lines (I_cal_) are calculated diffractograms, and the residual lines are shown in blue color. G represents goethite phase and Bragg’s peak positions are indicated with green vertical bars.

The average crystallite grain sizes and the parameters ε and σ were estimated by the three models (Figures S2–S4) and have shown similar values for the studied samples, as can be appreciated in Table S1. All these values differ from Scherrer’s estimation, and reinforce the idea of strain consideration in the estimation of crystallite grain sizes. One further model that contains the isotropic line broadening is the average size-strain model, where less weight is given to XRD data from Bragg peaks at high angles [27]. The model is represented by the following:

(14) $${\left(\frac{d_{hkl}\beta_{hkl}cos\theta }{\lambda }\right)}^{2}=\frac{K}{D}\left(\frac{d_{hkl}^{2}\beta_{hkl}cos\theta }{\lambda }\right)+{\left(\frac{\varepsilon }{2}\right)}^{2}$$

where *K* = 3/4 for spherical particles. A size-strain plot (SSP) of ${\left(\frac{d_{hkl}\beta_{hkl}cos\theta }{\lambda }\right)}^{2}$ versus $\left(\frac{d_{hkl}^{2}\beta_{hkl}cos\theta }{\lambda }\right)$ gave a particle size from the slope and the root of the y-intercept yields the strain that found that particle (see Figure S5). The R^2^ values are important to differentiate among all of the studied methods (see Table S1). We obtained only positive values of R^2^ for all of the crystallographic phases using the size-strain model, suggesting that this model better fits the XRD patterns presented in this study. To have a better understanding of the obtained fitted data, we used the advanced Rietveld method, the refined parameters of which are summarized in Table 1 for all samples. The pure zeolite Type 5A had a stoichiometric formula Ca_48_Al_96_Si_96_O_384_, which corresponds to a dehydrated Ca-A zeolite (a = 24.6 Å) [22]. Figure 1a shows the Rietveld refined diffractogram for this sample. A mean crystallite diameter of 380 nm was estimated. In contrast, the NPZEO1 and NPZEO2 samples (Figure 1b,c) display Bragg diffraction peaks in the region from 2 to 80 degrees, corresponding to inverse spinel cubic structure of the γ-Fe_2_O_3_ phase [29]. However, additional Bragg peaks with minor contributions to the XRD pattern of the NPZEO1 sample are present and correspond to the goethite phase, according to our Rietveld analysis.

**Figure 2:**
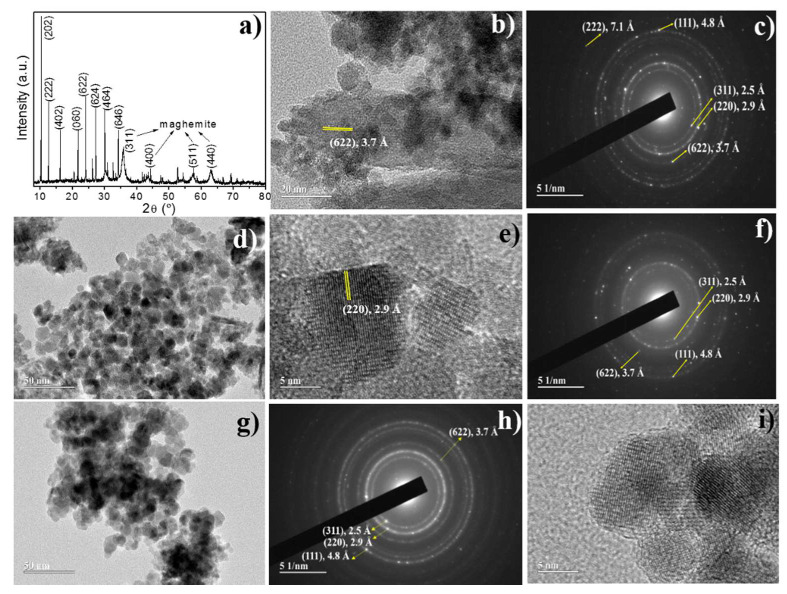
XRD pattern for the NPZEO3 composite, where the Miller indices are also indicated (**a**). Transmission electron microscopy (TEM) images for the NZPEO1 (**b**), NPZEO2 (**d**,**e**), NPZEO3 (**g**,**i**), and their respective selected area electron diffraction (SAED) patterns (**c**,**f**,**h**).

For the NPZEO1 sample, the Rietveld quantitative analysis of the identified crystal phases in the composites gave percentage values of 76% and 24% for γ-Fe_2_O_3_ and goethite, respectively. The presence of the goethite can be explained by the alkaline base that was used in the synthesis, as previously reported [29]. In fact, the alkaline reactant can severely affect the presence of secondary phases, like iron hydroxides. Moreover, as already expected, no zeolite diffraction peaks were observed in XRD patterns owing to its low concentration (it was used in the synthesis of 0.5 g of zeolite when compared with the relative high amount of iron reactive). For the NPZEO2 sample, only diffraction peaks of the γ-Fe_2_O_3_ NPs were found in the XRD pattern (no trace of goethite was found), indicating that the NPZEO2 sample is a composite of γ-Fe_2_O_3_ NPs and zeolite Type 5A. The mean crystallite size, 〈D〉, estimated from the refinement for the γ-Fe_2_O_3_ phase, was 7.0(5) nm for the NPZEO1 and 11.0(5) nm for the NPZEO2. For the NPZEO3 sample (Figure 2a), a major contribution of zeolite (phase percentage of 63%) is measured in the XRD pattern, while γ-Fe_2_O_3_ Bragg diffraction peaks were observed as a secondary phase (phase percentage of 37%). A value of 〈D〉 = 7.8(5) nm was also found using the Scherrer formula, as described above.

The DOC values, estimated from XRD patterns, were 88%, 92%, 94%, and 89% for zeolite type 5A, NPZEO1, NPZEO2, and NPZEO3 samples, respectively. The DOC of pure zeolite did not vary when mixing with maghemite nanoparticles (NPZEO3). Maghemite nanoparticles (NPZEO2) have a high degree of crystallinity, where the presence of goethite in the NPZEO1 sample slightly changes the DOC value. Recent studies [24,30] correlate the effects of the DOC values with the reactivity of the adsorbate, in our case, Pb(II). However, the big amount of crystalline nanomaghemite, when compared with low dosage of zeolite used in synthesis of the NPZEO1 and NPZEO2 sample, reduces the reactivity of pure zeolite for Pb(II) uptake. Thus, as proved by quantitative Rietveld analysis, the balance content of zeolite and nanomaghemite will exhibit remarkable adsorption properties for Pb(II), as will be shown below.

**Figure 3:**
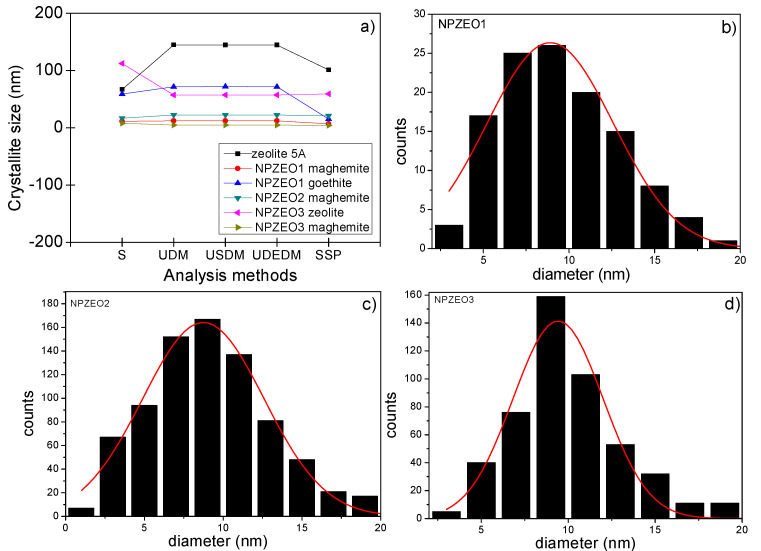
Crystallite size achieved by the analysis methods for all samples (**a**). Particle size distribution for the NPZEO1 (**b**), NPZEO2 (**c**), and NPZEO3 (**d**) samples.

The TEM images (Figure 2b–i) showed, in general, NPs with a broad size distribution with mean diameter sizes of 8.9(3) nm, 8.7(3) nm, and 9.4(3) nm for the NPZEO1, NPZEO2, and NPZEO3 samples, as obtained from pore size distribution fitted to a Gaussian profile (Figure 3b–d). These values differ from those obtained by the previous crystallite analysis methods (Figure 3a), because they do not consider the anisotropic line broadening often found in NPs [31], as the Rietveld method takes into consideration. From the zoomed images (Figure 2e,i), we can see that maghemite NPs form aggregates with zeolites. Moreover, the selected area electron diffraction (SAED) patterns (Figure 2c,f,h) confirm the presence of zeolite in the nanocomposite owing to the interplanar atomic distance of *d*_(622)_ = 3.7 Å and *d*_(222)_ =7.1 Å found in all samples and in agreement with the XRD pattern of zeolite (Figure 1a). The interplanar distances for maghemite phase were found to be *d*_(220)_ = 2.9 Å, *d*_(111)_ = 4.8 Å, and d_(311)_ = 2.5 Å, respectively.

### 3.2. Surface and Textural Properties

**Figure 4:**
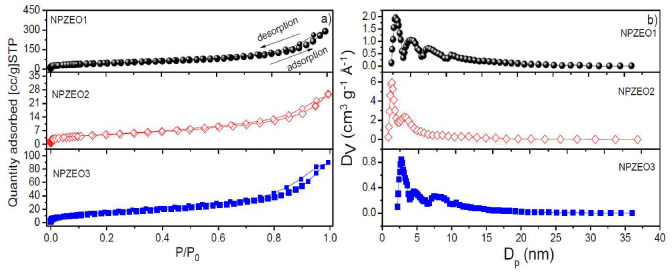
N_2_ adsorption–desorption isotherms (**a**) and pore size distribution (PSD) (**b**) for NPZEO1, NPZEO2, and NPZEO3 samples.

N_2_ adsorption–desorption isotherms determined at 77 K and pore size distributions, calculated by the NLDFT method, of magnetic zeolite nanocomposites are shown in Figure 4a,b, respectively. The textural properties, including specific BET surface area, pore volume, and pore width, are given in Table 2. The isotherms are classified as type IV, according to International Union of Pure and Applied Chemistry (IUPAC) classification for mesoporous adsorbents [32]. The γ-Fe_2_O_3_ NPs (9 nm in size) often present a low BET surface area of 88 m^2^/g [29]. So, in our case, we observed an enhancement of surface area up to 194 m^2^/g; an effect that can be explained by the presence of zeolite type 5A (571 m^2^/g). As a consequence, it gives more available sites to the whole material to adsorb and remove the lead cations. It is worth mentioning that the high value of surface BET area of the zeolite 5A is related to its microporosity (0.18 cm^3^/g) and additional mesoporosity (0.06 cm^3^/g), as indicated in Table 2 [33]. The values of pore volume (~0.35 to 0.40 cm^3^/g) for the NPZEO1 and NPZEO2 samples are similar to those found in graphene matrix loading high iron oxide NPs (ca. 6–10 nm) [34], with relative amounts of iron precursors used in the synthesis. The amount of adsorption for the NPZEO2 sample is significantly lower than those for the other two samples, but it is not reflected in the pore volume of the NPZEO2 sample. This effect may be related to the blocking of pore entrance or reduction of the free volume inside pores. Additionally, the NPZEO3 sample exhibited less pore volume (0.13 cm^3^/g), close to the pure zeolite, confirming the major contribution of zeolite on this sample.

### 3.3. Magnetic Properties

**Figure 5:**
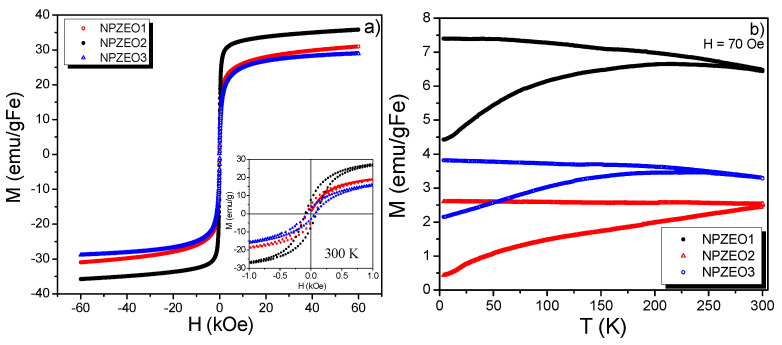
M-H loops recorded at 300 K (**a**) and zero field cooling (ZFC) and field cooling (FC) measurements (**b**) for NPZEO1, NPZEO2, and NPZEO3 samples. The inset in (**a**) is a magnification of the M-H loop in the region −1 to +1 kOe.

The M-H loops for the NPZEO1, NPZEO2, and NPZEO3 samples are shown in Figure 5a. The saturation magnetizations (M_s_) at RT for the NPZEO1 and NPZEO3 samples have values of ~30 emu/gFe, while the NPZEO2 sample has 36 emu/gFe; that is, M_s_ values of our samples are smaller than 60 emu/gFe reported for pure γ-Fe_2_O_3_ NPs [29]. The M_s_ value reduction is also consistent with their particle size distributions, as shown by their TEM histograms (the three samples have close particle sizes between 8 and 9 nm). In addition, different experimental methods result in different particle morphologies, so, a good explanation for a reduction of M_s_ relative to the pure γ-Fe_2_O_3_ NPs is the enhancement of the surface magnetic disorder of our samples and, consequently, low magnetization owing to magnetic frustration (magnetic dead-like layer) [29,31]. The remanent moments have values of 4.3 (3), 7.6 (3), and 3.7 (3) emu/g and the coercive fields have close values of 260 (5) Oe for all the samples. The thermal dependence of the susceptibility M-T is given in Figure 5b. The ZFC and FC signals revealed a magnetic blocked behavior near RT for the three samples. The shapes of M-T curves (ZFC and FC) suggest that our samples have a broad particle distribution, as observed by TEM images, and as expected owing to the chemical co-precipitation method. The nanocomposites were tested under applied field to guarantee that they are suitable for magnetic remediation, as a magnetic dead-layer could reduce its applicability, and we observed that the nanocomposites are well sensitive to the external field yielded by permanent magnets. The magnetic properties presented by the samples guaranteed a good magnetic removal performance, as will be discussed in the next section.

### 3.4. Effect of Variable Parameters on the Pb (II) Adsorption Process

**Figure 6:**
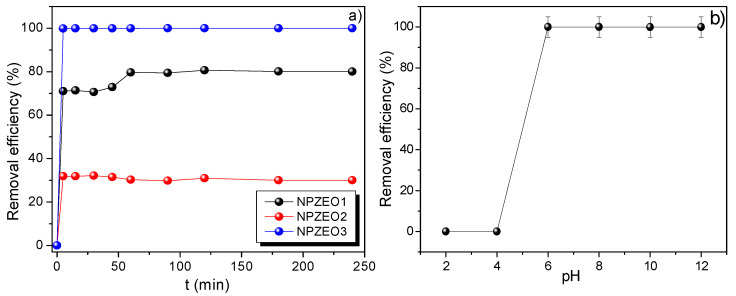
Pb(II) adsorption kinetic curves obtained for NPZEO1, NPZEO2, and NPZEO3 samples (**a**). pH dependence of the removal efficiency for sample NPZEO3 (**b**).

Before conducting in detail adsorption studies, the adsorption kinetic performance at pH = 6 and 300 K was tested for the three synthesized samples (see Figure 6a) with the aim of finding the highest removal efficiency among the studied adsorbents. The removal efficiency was calculated by the following:

(15) $$R\left(\%\right)=\frac{C_{0}-C_{f}}{C_{0}}\times 100\;\%$$

We checked that the NPZEO1 and NPZEO2 samples have shown less removal efficiency, as compared with the NPZEO3 sample; an effect that was mainly attributed to the high iron oxide content present in the first two samples. As our aim was to find an adsorbent with remarkable adsorption and magnetic separation properties, we selected the NPZEO3 sample for more detailed and systematic adsorption studies.

### 3.5. Effect of pH and Adsorption Mechanism

**Figure 7:**
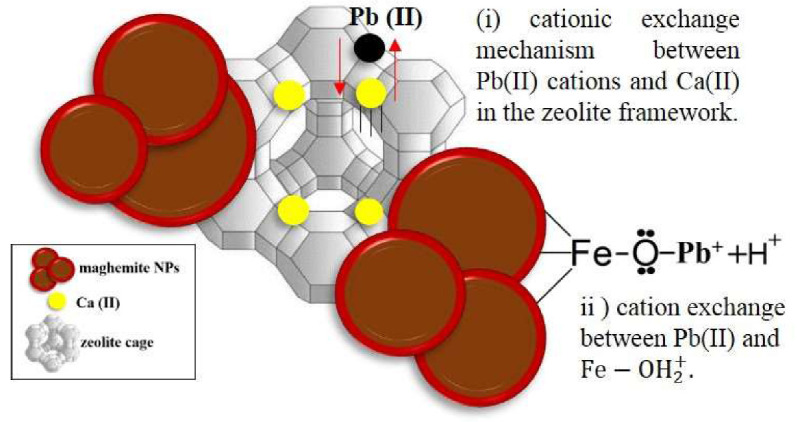
Proposed scheme to describe the mechanism for the Pb(II) adsorption using the NPZEO3 sample as adsorbent. Notice that maghemite nanoparticles (NPs) have a size distribution and sizes that are bigger than zeolite cages.

The pH dependence of the adsorbed amount is shown in Figure 6b. It can be clearly observed that the adsorption efficiency is sensitive to pH changes. Specifically, at low pH values (<4), the removal capacity of Pb(II) is relatively low. This can be explained if one considers repulsive electrostatic forces between the protonated surface of maghemite ($\mathrm{Fe}-{\mathrm{OH}}_{2}^{+}$) and the metal lead cations. The same tendency at low pH was also reported in recent works for divalent ions [16,35]. For pH > 5, the removal efficiency is significantly increased to 99.9%, a value that is kept unchanged for high alkaline intervals. At high pH values (~10), the NPs surfaces are coated with negative hydroxyl groups ($\equiv \mathrm{FeOH}⇌{\mathrm{FeO}}^{-}+H^{+}$), by which exchange of metal ions with H^+^ is favored at the NPs surface [10]. In addition, the zeolite type 5A can also play an important role in the adsorption enhancement owing to the cation exchange interaction between Pb^2+^ and Ca^2+^ in the dehydrated Ca-A zeolite framework. A representative scheme that describes the mechanism for the lead adsorption in our composites is given in Figure 7.

### 3.6. Effect of the Amount of NPZEO3 on the Adsorption Process

**Figure 8:**
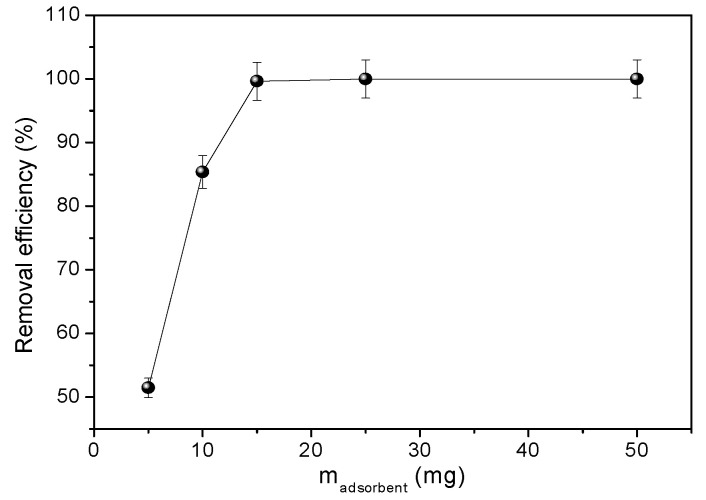
Adsorbent mass dependence of the removal efficiency *C*_0_ = 70 mg L^−1^.

The effect of the NPZEO3 dosage in the adsorbed amount was studied and the results are plotted in Figure 8. We can observe that the adsorbed amount increases with the increasing adsorbent mass and reaches a plateau at 15 mg. The agitation speed was another variable parameter studied in our work. We can clearly distinguish from Figure S6 that the removal efficiency of 100% is speed agitation independent, which is good for technological purposes, because in big baths, it is quite difficult to guarantee homogeneous agitation of an effluent.

### 3.7. Adsorption Kinetics

**Figure 9:**
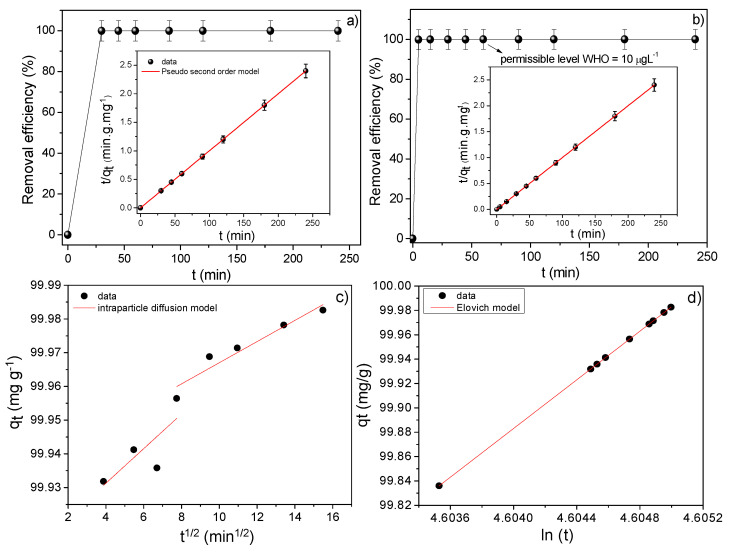
Time dependence of Pb(II) adsorption capacity for the zeolite type 5A (**a**) and NPZEO3 (**b**). The insets show the data fitted to the pseudo kinetic order model. Intraparticle diffusion model (**c**) and Elovich kinetic model (**d**) applied to the NPZEO3 sample are also displayed. WHO, World Health Organization.

Figure 9a,b show the adsorption kinetic profile of the zeolite type 5A and NPZEO3 for an interval of 5 min to 4 h. To decrease the level of pollution and to enhance potential applications of the materials, the tests of lead adsorption and magnetic removal must be done in a shortest time. From these kinetic curves, it can be noted that the equilibrium time is quickly reached in 5 min for the NPZEO3 sample. To our knowledge, this time is the smallest equilibrium time reported in the literature as compared with several systems given in Table 3.

In addition, our data have shown that the adsorbed amount reached a remarkable value of 99.99% in the first 5 min of contact time, with a reduction in the Pb(II) concentration from an initial concentration of 50 mg L^−1^ to 82 μg L^−1^. This demonstrates the high potential of our system to adsorb lead from Pb-contaminated water. For immersion times higher than 30 min, the concentrations are below the detection limit of ICP-OES equipment, 10.4 μg L^−1^, which is the permissible concentration of lead in drinking water, according to WHO guidelines. Zeolite type 5A is a remarkable adsorbent, as we demonstrated (after only 15 min, it adsorbed all lead from water). However, it is necessary to have an adsorbent that can be easily removed from water, which is why we form a hybrid with maghemite to combine both adsorption and removal magnetic separation properties. The cost of combining both phases is a reduction in the kinetic rate constant, but it is still faster than other parent systems (Table 3), which are dominated by a slow *k*_2_ value. The use of magnetic particles helps the entire process and improves technological applications where contaminants should be completely removed from the effluents (zeolites that are not removed will become a contaminant as well). Further, the adsorption kinetic behavior was studied using the following kinetic adsorption models given by Equations (16)–(18) [39]:

(16) $$\frac{t}{q_{t}}=\frac{1}{k_{2}q_{e}^{2}}+\frac{t}{q_{e}}\;(\mathrm{Pseudo}\;\mathrm{second}\;\mathrm{order}\;\mathrm{model})$$

(17) $$q_{t}=k_{p}t^{1/2}+C\;\left(\mathrm{intraparticle}\;\mathrm{diffusion}\;\mathrm{model}\right)$$

(18) $$q_{t}=\frac{1}{\beta }\ln\left(\alpha \beta \right)+\frac{1}{\beta }ln\left(t\right)\;\left(\mathrm{Elovich}\;\mathrm{kinetic}\;\mathrm{model}\right)$$

where $k_{2}$ (g mg^−1^ min^−1^) is the pseudo-second-order rate constant and $k_{2}q_{e}^{2}$ is the initial adsorption rate, $k_{p}$ relates the rate constant of intra-particle diffusion (mg g^−1^ m^−0.5^), C corresponds to the boundary layer thickness, *α* is the initial adsorption rate (mg min^−1^) at *t* = 0, and β represents the surface coverage length and activated energy (g mg^−1^). By fitting the straight line of $\frac{t}{q_{t}}$ versus *t*, values of $\frac{1}{k_{2}q_{e}^{2}}$ as the intercept and $\frac{1}{q_{e}}$ as the slope were obtained (insets of Figure 9a,b). The value of $q_{e}$ was found to be equal to 100 mg g^−1^, which is identical to the experimental one. The R^2^ parameter had a value of 0.995, which suggests that the kinetic adsorption process is governed by chemisorption of lead on zeolite type 5A and in the composite. Moreover, the value of *k*_2_ was equal to 35.5 and 0.9 g mg^−1^ min^−1^ for the zeolite type 5A and NPZEO3 samples, respectively. The obtained values are bigger when compared with the parent systems used for lead adsorption, as shown in Table 3. When using the intraparticle diffusion model for the NPZEO3 sample, a two-step kinetic adsorption behavior is observed. The first step corresponds to the diffusion of Pb(II) through the solution to the adsorbent surface, whereas the second step is a slow adsorption stage related to intraparticle diffusion of Pb(II) cations through adsorbent’s pores (in this case, the mesoporous of zeolite and maghemite). The obtained parameters, calculated by fitting Figure 9c, gave values of $k_{p1}$ = 3 × 10^−3^ mg g^−1^ m^−0.5^, *C*_1_ = 99.94 mg g^−1^, R^2^ = 0.90 and $k_{p2}$ = 5 × 10^−3^ mg g^−1^ m^−0.5^, *C*_2_ = 99.91 mg g^−1^, R^2^ = 0.90. The plot (Figure 9d) of *q_t_* versus ln*t* has a good linear behavior with R^2^ = 0.999. This means that the Elovich kinetic model fits well the adsorption data, in agreement with the pseudo-second-order model, that is, it confirms a chemisorption process. To find the best model that describes our kinetic adsorption data, we employed the AIC calculation given by Equation (2) [26], using the values of the Table 4, and we conclude that the intraparticle diffusion model better describes the kinetic adsorption behavior of Pb(II) onto the NPZEO3 surface. In this regard, the intraparticle diffusion kinetic model is preferable especially when using hybrids of porous/mesoporous material such as zeolite and maghemite NPs.

### 3.8. Adsorption Isotherm

**Figure 10:**
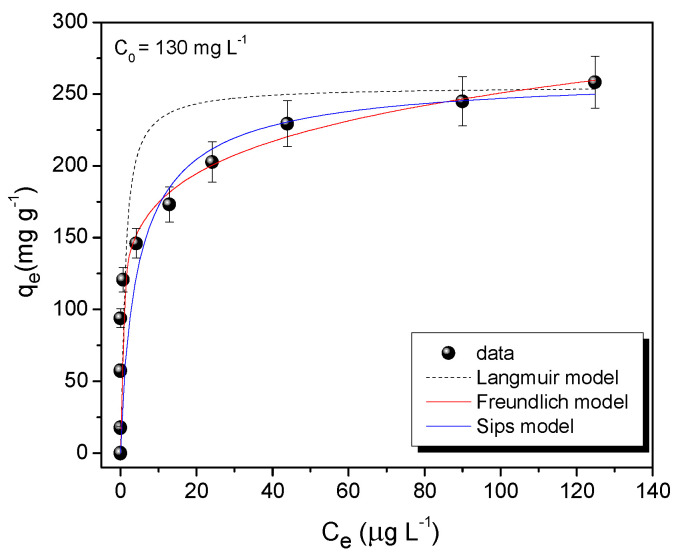
Pb(II) adsorption isotherm for NPZEO3 as adsorbent in Pb-contaminated water.

Figure 10 displays the adsorption isotherm obtained for the NPZEO3 sample at pH = 5.5. Three adsorption models [40] were used to study the adsorption process performed at the composite surface:

(19) $$q_{e}=\frac{Q_{0}bC_{e}}{1+bC_{e}}\;(\mathrm{Langmuir}\;\mathrm{model})$$

(20) $$q_{e}=K_{F}C_{e}^{1/n}\;(\mathrm{Freundlich}\;\mathrm{model})$$

(21) $$q_{e}=\frac{q_{m_{S}}k_{S}C_{e}^{m_{S}}}{1+k_{S}C_{e}^{m_{S}}}\;(\mathrm{Sips}\;\mathrm{model})$$

where $q_{e}$ is the amount of adsorbate adsorbed per unit weight of adsorbent, $C_{e}$ (μg L^−1^) is the equilibrium adsorbate concentration in the final solution, $Q_{0}$ is the maximum adsorption capacity (mg g^−1^), b (mg^−1^ L) is the Langmuir constant, $K_{F}$ (mg^1−n^ L^n^/g) and 1/n are assigned to the adsorption capacity and strength, $q_{m_{S}}$ (mg g^−1^) is the Sips maximum adsorption capacity, $k_{S}$ (L mg^−1^)^m^ the Sips equilibrium constant, and m_S_ is the Sips model exponent. The parameters obtained after fitting the isotherm data are summarized in Table 5. It can be seen that the Langmuir model gave a value of $Q_{0}$ = 252 (5) mg g^−1^, which is a high value when compared with those reported in the literature [16,17,36].

However, the small value of R^2^ = 0.88 suggests that the adsorption process is not governed by monolayer covering. This is correlated to the non-saturation behavior in the high concentration range. Looking to the fitting parameters, obtained by the Freundlich and Sips methods, we concluded that the adsorption process occurs over a heterogeneous surface, where a finite maximum adsorption capacity value is not reached. The increment in the $q_{m_{S}}$ value, as compared with the value from the Langmuir method, suggests that our system can work successfully in higher Pb concentration ranges, providing wider usability for our magnetic composite adsorbent. The estimated AIC values of 27.9 and 31.9 for Sips and Langmuir models reinforce that the Sips model is the best model to describe our experimental data.

### 3.9. Interferent Ions and Reusability

**Figure 11:**
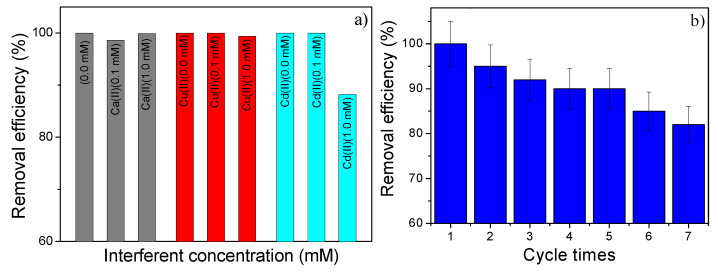
Effect of interfering divalent cations on the removal efficiency percentage (**a**) and recycling performance of the NPZEO3 sample as absorbent (**b**). Pb(II) initial concentration = 70 mg L^−1^.

A water sample under realistic conditions (effluents from industries, for example, mining tailings) presents additional divalent cations that can significantly affect the performance of the adsorbent to remove Pb(II). Therefore, adsorption experiments in the presence of other divalent cations, like Ca(II), Cu(II), and Cd(II), were also studied, and the results are shown in Figure 11a. It can be seen that the removal efficiency is not affected by the use of 0 to 1.0 mM of Ca(II) and Cu(II). However, the use of Cd (1.0 mM) slightly decreases the removal efficiency from 99.9% to 88.2%. To test the reuse of produced magnetic adsorbent of this work, we performed experiments (the results are shown in Figure 11b) with following the steps: after reaching the equilibrium time, the condensed material was separated by filtration and magnetic decantation and led to a treatment with 0.1 M HCl for Pb(II) desorption. Thereafter, repeated adsorption experiments were performed using an initial concentration of 70 mg L^−1^. Seven cycles were tested and the adsorption removal efficiency remained at about 82%. Our results clearly demonstrate that the adsorption is quite stable and that the adsorbent NPZEO3 has remarkable power to be used more than once in a clear water process, that is, the results suggest that these materials have enhanced capabilities for technological applications as a magnetic remediation absorbent nanocomposite.

## 4. Conclusions

Using the alkaline co-precipitation route, nanocomposites of maghemite and zeolite type 5A were successfully synthesized and used for the Pb(II) adsorption and magnetic removal process. Among the three synthesized systems with grain sizes from 7 to 11 nm, the NPZEO3 sample showed the best adsorption performance with 99.99% removal efficiency. The adsorption kinetic experiments for the NPZEO3 sample show an enhanced equilibrium time of 5 min, where 50 mg L^−1^ Pb(II) is totally removed from water by a permanent magnet that does not affect the magnetic response of the NPZEO3 sample. Further, the effects of variable parameters like adsorbent mass, agitation speed, and pH were systematically studied. Changes in the pH parameter allowed the study of the adsorption mechanism, where a cation exchange interaction was favored owing to the presence of Ca(II) in the zeolite framework and surface chemical configuration of the maghemite. Additionally, the adsorption isotherm data follow the Sips model, revealing that the system can be used for high Pb(II) concentrations, with an extrapolated value of 265 mg g^−1^ for 130 mg L^−1^ of initial concentration. Besides that, our sample revealed remarkable adsorption properties in the presence of interferent cations, such as Ca(II), Cu(II), and Cd(II). It has also been demonstrated that, after seven regeneration cycles, the efficiency for lead removal of the NPZEO3 sample was kept at 82%, that is, it has excellent properties for the adsorption and removal of Pb(II). All three nanocomposites presented in this work are promising as adsorbent materials for water remediation processes.

## 5. Patents

The presented work is under request of patent with number 001335-2019/DIN, Indecopi-Peru.

## Figures and Tables

**Table 1:** Rietveld refinement parameters of zeolite type 5A, NPZEO1, and NPZEO2 samples using the Fullprof program: cell parameters, cell volume, global average size, and agreement factors. *R_p_* (%) and *R_wp_* (%) are the profile residual and the weighted profile residual factors, respectively, used to verify the Rietveld refinement quality. The goodness of fit, *χ*^2^, was equal to ${\left(\frac{R_{wp}}{R_{exp}}\right)}^{2}$, where *R_exp_* is the expected profile residual.

Refined Parameters	Zeo5A	NPZEO1		NPZEO2
		γ-Fe_2_O_3_	goethite	γ-Fe_2_O_3_
a (Å)	24.5586	8.3506	9.9182	8.3022
b (Å)	24.5586	8.3506	3.0059	8.3022
c (Å)	24.5586	8.3506	4.5801	8.3022
α (°)	90	90	90	90
β (°)	90	90	90	90
γ (°)	90	90	90	90
V (Å^3^)	14,811.94	582	136.5	572
global average size (nm)	373	6.6	290	10.5
*R_p_* (%)	16.9	55		53
*R_wp_* (%)	19.1	43.8		35.7
*R_exp_* (%)	16.8	24.8		27.9
χ^2^	1.3	3.1		1.6

**Table 2:** Textural parameters obtained for zeolite Type 5A, NPZEO1, NPZEO2, and NPZEO3 adsorbents. BET, Brunauer–Emmett–Teller.

Adsorbent	BET Surface Area (m^2^/g)	Pore Volume (cm^3^/g)	Pore Width (nm)
zeolite type 5A [33]	571	0.18	0.5
NPZEO1	167	0.40	3.2
NPZEO2	178	0.35	1.4
NPZEO3	194	0.13	2.8

**Table 3:** Adsorption kinetic parameters of several systems found in the literature given for comparison, where *t* is the equilibrium time for adsorption. NPs, nanoparticles.

Adsorbent	*k*_2_ (mg g^−1^ min^−1^)	R (%)	*t* (min)	*C*_0_ (mg L^−1^)	Dose (g L^−1^)
Co-Fe_2_O_3_ [16]	1.74	95	45	20	0.1
ZIF-8@GO [35]	5.89 × 10^−3^	-	100	30	0.15
MNPs@carboxymethylated biopolymers [17]	34 × 10^−3^	99.9	180	24	2
nanoscale zero-valent irons (nZVI) [36]	7.65 ×10^−4^	~90	200	100	0.35
α-Fe_2_O_3_ NPs [37]	12 ×10^−4^	~88	240	10	0.1
Fe_3_O_4_@SBA-15 [38]	11 × 10^−3^	-	720		
zeolite 5A (this work)	35.5	100	15	50	0.5
NPZEO3 (this work)	0.9	100	5	50	0.5

**Table 4:** Values of Akaike’s information criterion (AIC) for the three kinetic adsorption models used in this work.

Model	*q_t,exp_*	*q_t,model_*	*AIC*
Pseudo second order model	99.8	100	−50.12
Intraparticle difusion model	99.8	99.91	−40.28
Elovich	99.8	99.908	−46.73

**Table 5:** Parameters obtained from fitting the Langmuir, Freundlich, and Sips models to the experimental isotherm for Pb(II) adsorption.

Pb (II) Isotherm		
Langmuir Parameters	Freundlich Parameters	Sips Parameters
*Q_0_* (mg g^−1^) 252 (5)	*K_F_* (mg g^−1^) 122 (5)	$q_{m_{S}}$ (mg g^−1^) 265 (5)
*b* (mg^−1^ L) 0.99 (1)	1/*n* 0.16 (5)	$k_{S}$ (L mg^−1^)^m^ 0.25 (5)
R^2^ 0.88	R^2^ 0.97	$m_{S}$ 0.87 (5)
		R^2^ 0.98

## Supplementary Materials

The following are available online at https://www.mdpi.com/2079-4991/10/9/1668/s1, Figure S1: Scherrer plot; Figure S2: W–H plot; Figure S3: modified W–H plot; Figure S4: modified W–H plot; Figure S5: SSP plot; Figure S6: Speed agitation dependence of the removal efficiency; Table S1: Microstructural parameters.
