# Supplementary material for: Improved Removal Capacity and Equilibrium Time of Maghemite Nanoparticles Growth in Zeolite Type 5A for Pb(II) Adsorption
Source: Nanomaterials (Basel). 2020 Aug 26;10(9):1668. doi: 10.3390/nano10091668 (PMC7558356; doi:10.3390/nano10091668)
Supplement: Supplementary file 1 [file nanomaterials-10-01668-s001.pdf]

Supplementary materials

Table S1. Microstructural parameters obtained by several crystalline size analysis methods and TEM.

samples	Scherrer's method		Williamson-Hall method												size- strain method			TEM		
			UDM			USDM				UDEDM										
	D (nm)	R^2	D (nm)	ε $(\times 10^{-3})$	R^2	D (nm)	ε $(\times 10^{-3})$	σ (MPa)	R^2	D (nm)	ε $(\times 10^{-3})$	σ (MPa)	U $(kJ\ m^{-3})$	R^2	D (nm)	ε (10^{-3})	R^2	D (nm)	σ (MPa)	R^2
**Zeolite 5A (pure)**	66.9	0.9547	144.7	1.6	0.9759	144.6	1.6	299.0	0.9759	144.6	1.6	299.0	238.4	0.97588	101.1	665.70	0.9953	-	-	-
**maghemite NPZEO1**	11.2	0.1667	12.2	1.5	-0.139	12.2	1.5	275.5	-0.139	12.2	1.5	275.5	202.4	-0.1388	6.9	2374.3	0.9862	8.9	3.7	0.9559
**goethite NPZEO1**	58.9	- 0.058	71.6	3.7	-0.019	72.0	3.7	684.3	-0.019	71.6	3.7	685.7	1254.4	-0.0188	15.3	159.5	0.6546	-	-	-
**maghemite NPZEO2**	16.8	0.7502	22.3	1.3	0.5810	22.3	6.4	251.7	0.5810	22.3	1.3	251.7	168.9	0.58101	20.9	954.6	0.9902	8.7	3.9	0.9670
**zeolite 5A NPZEO3**	112.3	-0.041	57.3	1.1	-0.200	57.3	1.1	207.3	-0.200	57.3	1.1	204.4	111.4	-0.2017	59.2	452.4	0.7866	-	-	-
**maghemite NPZEO3**	7.8	-0.489	4.8	-5.7	-0.384	4.8	5.7	1060.0	-0.383	4.8	5.7	1065.5	3027.6	-0.0383	4.1	3863.8	0.8080	9.4	2.6	0.9372

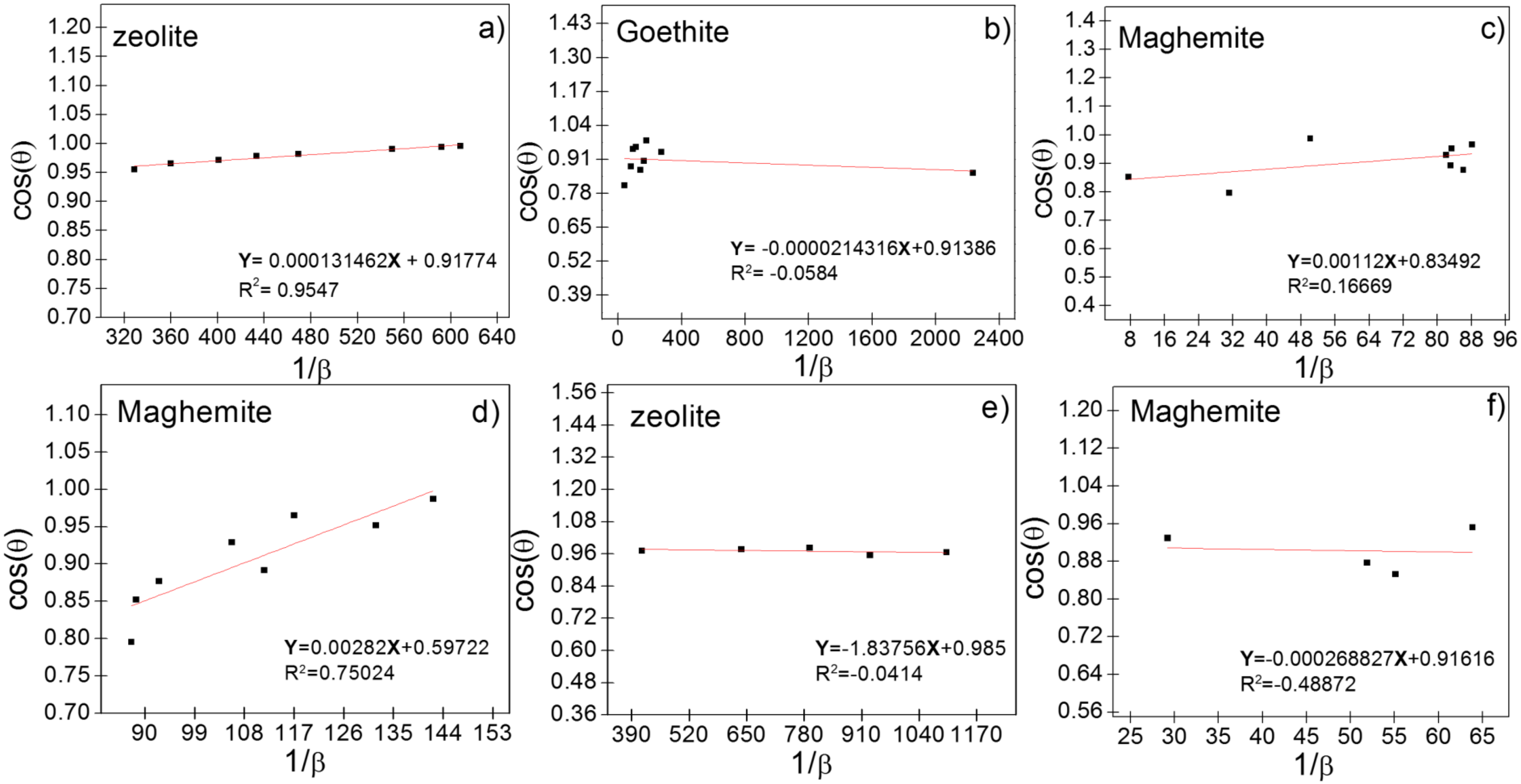

Figure S1. Scherrer plot of pure zeolite (a), NPZEO1 (b,c), NPZEO2 (d) and NPZEO3 (e,f). The average crystallite size is obtained from the slope of fitting.

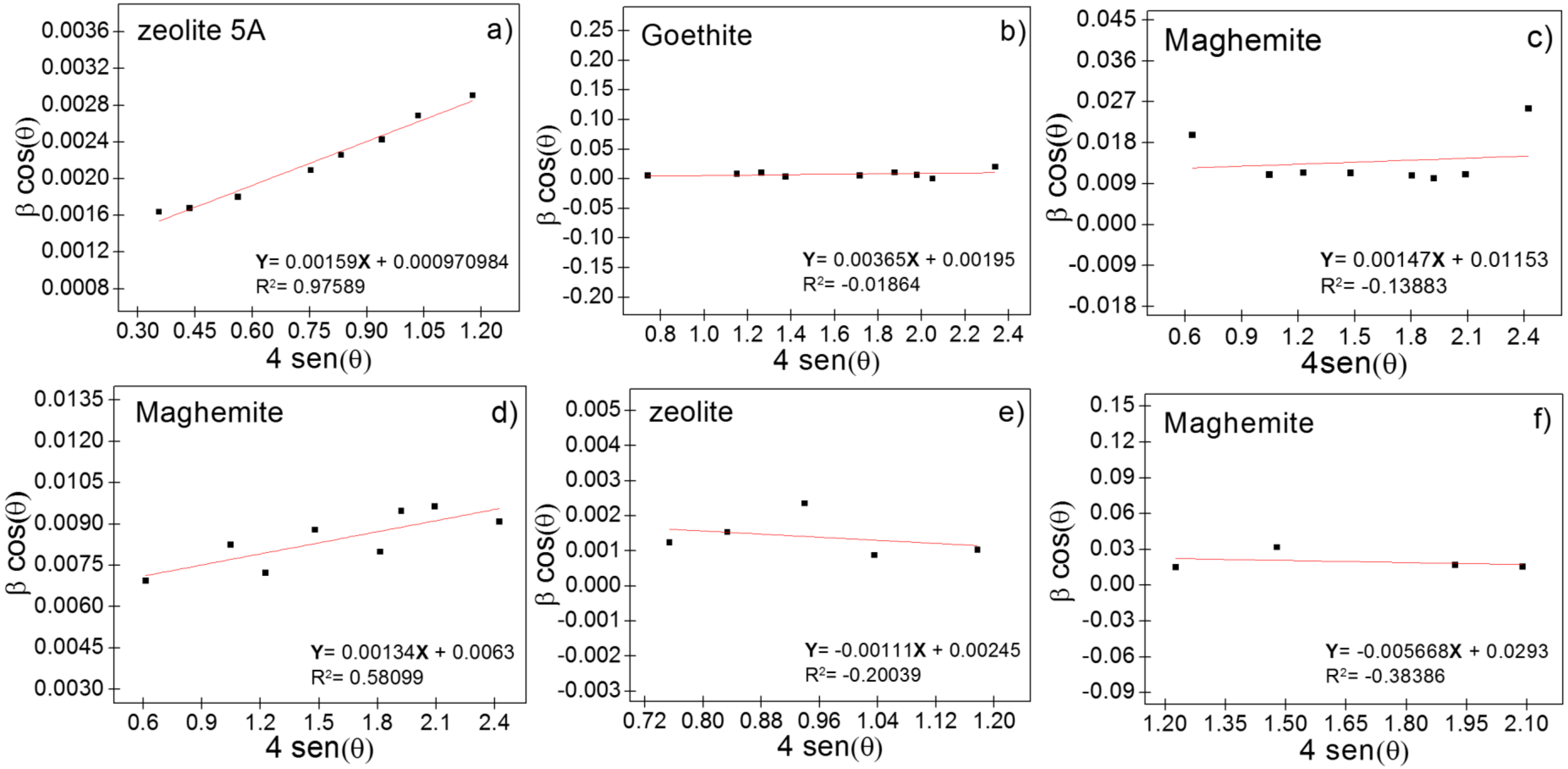

Figure S2. The W-H analysis of pure zeolite (a), NPZEO1 (b,c), NPZEO2 (d) and NPZEO3 (e,f) assuming UDM. The strain is obtained from the slope and the average crystalline size from the y-intercept of linear fitting.

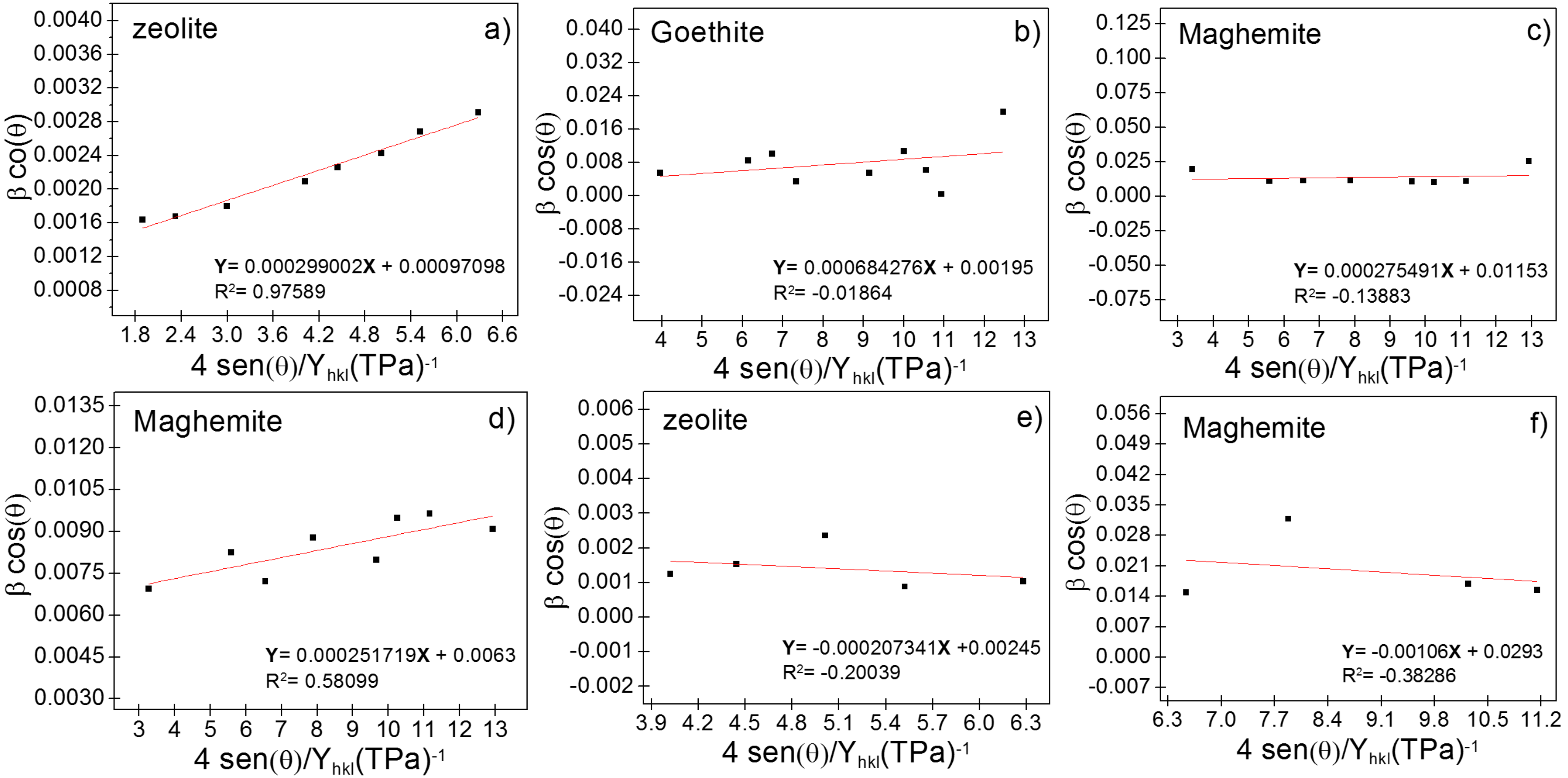

Figure S3. The modified W-H analysis of pure zeolite (a), NPZEO1 (b,c), NPZEO2 (d) and NPZEO3 (e,f) assuming USDM. The strain is obtained from the slope and the average crystalline size from the y-intercept of linear fitting.

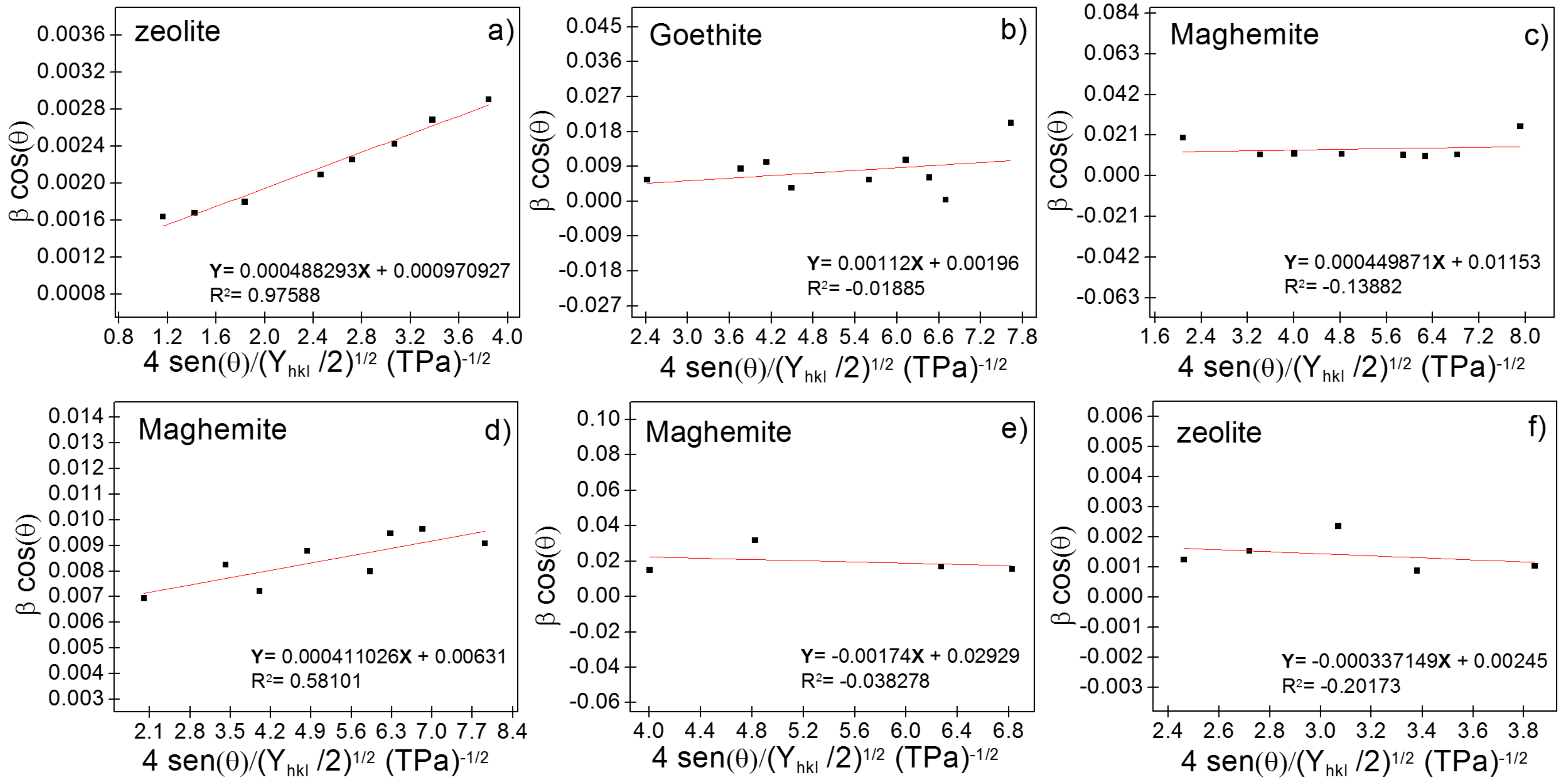

Figure S4. The modified W-H analysis of pure zeolite (a), NPZEO1 (b,c), NPZEO2 (d) and NPZEO3 (e,f) assuming UDEDM. The density of energy is obtained from the slope and the average crystallite size is obtained from the y-intercept of the linear fitting.

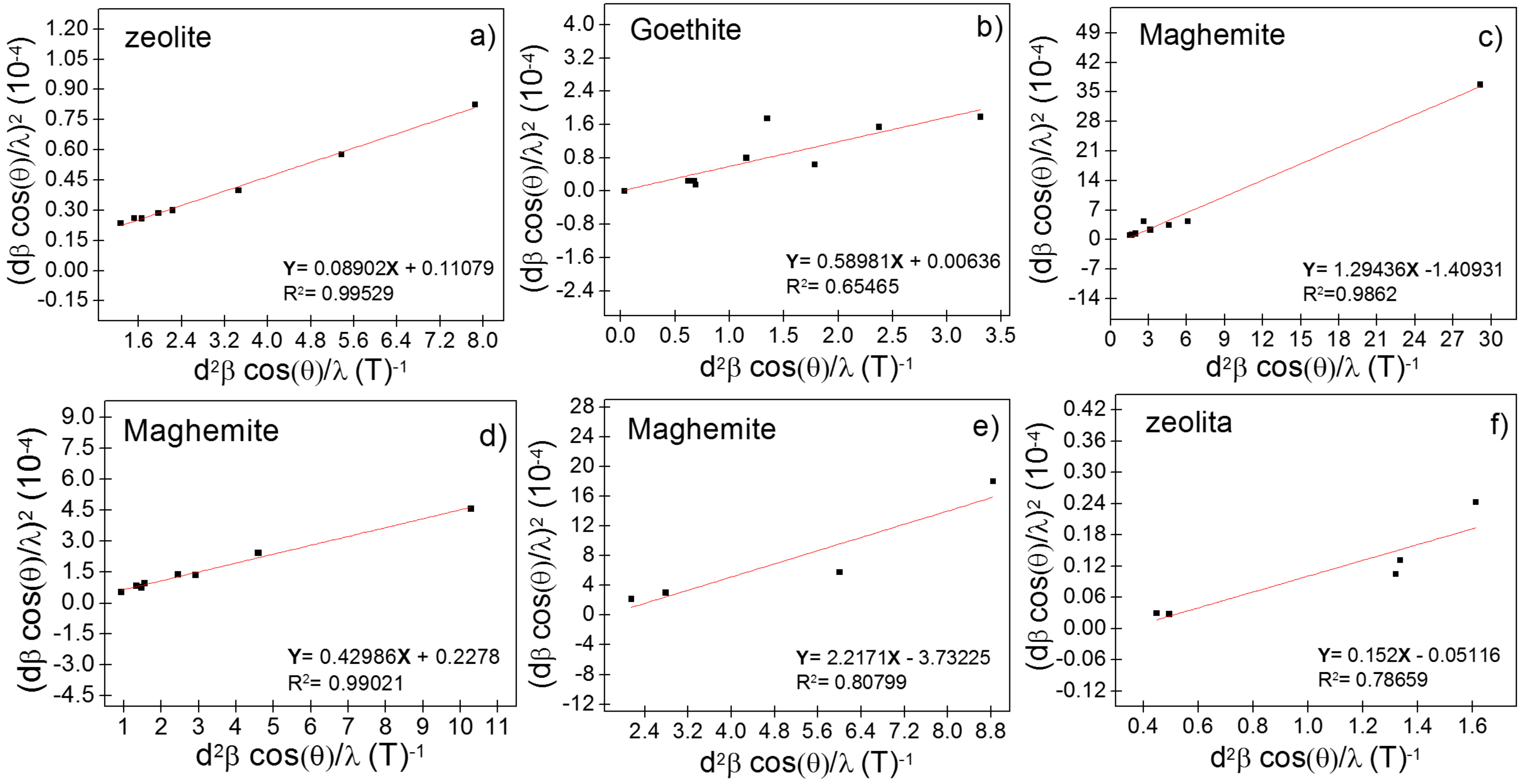

Figure S5. The SSP of pure zeolite (a), NPZEO1 (b,c), NPZEO2 (d) and NPZEO3 (e,f). The particle size is obtained from the slope of linear fitting and the root of y-intercept reveals the strain.

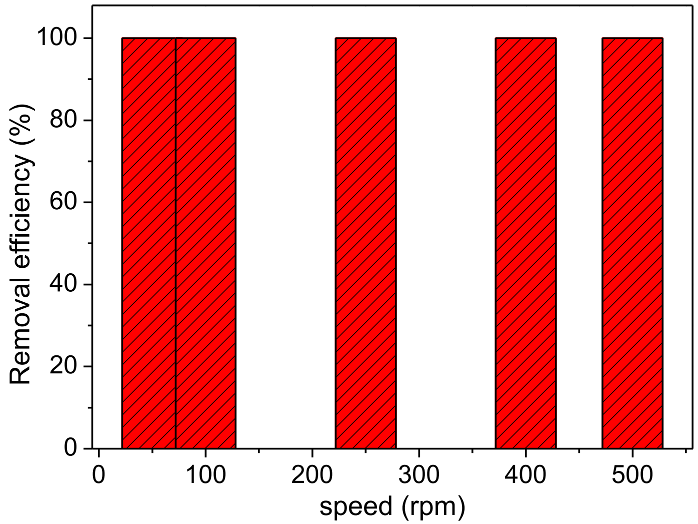

Figure S6. Speed agitation dependence of the removal efficiency.
